# A New Method for Accelerated Aging of Nanoparticles to Assess the Colloidal Stability of Albumin-Coated Magnetic Nanoparticles

**DOI:** 10.3390/nano15070475

**Published:** 2025-03-21

**Authors:** Boris Nikolaev, Ludmila Yakovleva, Viacheslav Fedorov, Natalia Yudintceva, Daria Tarasova, Elizaveta Perepelitsa, Anastasia Dmitrieva, Maksim Sulatsky, Sivaprakash Srinivasan, Shirish H. Sonawane, Anusha Srivastava, Sharad Gupta, Avinash Sonawane, Stephanie E. Combs, Maxim Shevtsov

**Affiliations:** 1 Laboratory of Biomedical Nanotechnologies, Institute of Cytology of the Russian Academy of Sciences (RAS), 194064 Saint Petersburg, Russia; nikolaevhpb@gmail.com (B.N.); yakluda5@gmail.com (L.Y.); gotosethxen@hotmail.com (V.F.); yudintceva@mail.ru (N.Y.); dariyataras0va@yandex.ru (D.T.); dmitriyeva03@bk.ru (A.D.); m_sulatsky@mail.ru (M.S.); 2 Personalized Medicine Centre, Almazov National Medical Research Centre, 197341 Saint Petersburg, Russia; 3 Department of Inorganic Chemistry and Biophysics, Saint-Petersburg State University of Veterinary Medicine, 196084 Saint-Petersburg, Russia; 4 Research Center of Neurology, 125367 Moscow, Russia; ross8marquise@gmail.com; 5 Department of Chemical Engineering, National Institute of Technology, Warangal 506004, Telangana State, India; ss21chrer06@student.nitw.ac.in (S.S.); shirish@nitw.ac.in (S.H.S.); 6 Department of Biosciences & Biomedical Engineering, Indian Institute of Technology Indore, Indore 453552, Madhya Pradesh, India; phd2201171001@iiti.ac.in (A.S.); shgupta@iiti.ac.in (S.G.); asonawane@iiti.ac.in (A.S.); 7 Department of Radiation Oncology, Technische Universität München (TUM), Klinikum Rechts der Isar, 81675 Munich, Germany; stephanie.combs@tum.de

**Keywords:** metal oxide nanoparticles, superparamagnetic iron oxide nanoparticles, SPIONs, protein corona, nanoparticle aggregation, method of accelerated aging, nanoparticle stability

## Abstract

The colloidal long-storage stability of nanosized drugs is a crucial factor for pharmacology, as they require much time for robust estimation. The application of bioavailable magnetic nanosuspensions in theranostics is limited by incomplete information about their colloidal stability in the internal media of human organisms. A method for the accelerated temperature stress “aging” of magnetic nanosized suspensions is proposed for the rapid assessment and prediction of the colloidal stability over time of nanosized iron oxide suspensions stabilized by albumin HSA. Colloidal stability is assessed using dynamic light scattering (DLS), fluorescence spectroscopy, electrophoresis, and ion monitoring methods during short- and long-term storage. Rapid assessment is achieved by short high-temperature (70 °C) processing of carboxymethyl-dextran-coated nanosol in the presence of albumin. The role of albumin in the sustained stability of superparamagnetic iron oxide particles (SPIONs) was studied under conditions mimicking blood plasma (pH = 7.4) and endolysosomal cell compartments (pH = 5.5). According to the fluorescence quenching and DLS data, colloidal stability is ensured by the formation of an HSA corona on carboxymethyl-dextran-coated SPIONs and their process of clustering. In the presence of albumin, the colloidal stability of nanoparticles is shown to increase from 80 to 121 days at a storage temperature of 8 °C The prognostic shelf life of magnetic nanosol is estimated by calculating the Van’t Hoff’s relation for the rate of chemical reactions. The validity of using the Van’t Hoff’s rule is confirmed by the agreement of the calculated activation energy at 8 °C and 70 °C. The developed method of the accelerated aging of nanoparticles can not only be employed for the estimation of the shelf life of magnetic nanoparticles coated with HSA in vitro but also for assessing the stability of SPIONs applied in vivo.

## 1. Introduction

Magnetic nanoparticles (MNPs) are nanoscale materials used in analytical chemistry and biosensors. They consist of a metal core and an outer shell, with the number of atoms varying from a few units to several thousand [1,2,3,4]. These nanoclusters, with their active surface and high sorption capacity, can interact with various biomolecules because of their small size. They also have ferro- and superparamagnetic properties, making them useful in precision medicine [5,6]. A variety of magnetic nanoparticles have been synthesized, including Co, Fe, Ni, iron oxides, and ferrites. Superparamagnetic iron oxide nanoparticles (SPIONs), with the formula Fe_3_O_4_, are particularly of interest for research and application in biomedicine because of their cost-effectiveness, stability, and biocompatibility [7,8,9]. Using various synthesis and processing methods, monodisperse suspensions and other magnetic nanoparticles with varying surface morphologies can be obtained [10,11,12]. For the biomedical application of nanoparticles, it is necessary that they fulfill a number of requirements, including the ability to form a stable colloidal system in aqueous solutions and other biocompatible solvents and to vary the parameters of the solution (e.g., salt concentration, pH, and temperature) within intervals determined by the specific purpose of the study. However, because of their high reactivity, there is practically no inert medium for nanoparticles. One of the key features of nanoparticles in solution is their tendency to aggregate; therefore, the practical use of nanoparticle solutions is associated with their stabilizing agents (coating the surface of the magnetic “core”, adding stabilizers, selecting solvents, etc.) [13].

The stability of a colloidal solution includes both its aggregate and kinetic consistency. Aggregative stability is the immutability of particle sizes in a colloidal solution over time, preventing particles from sticking together, which is considered to be more important for system applications. Sedimentation stability refers to the uniform distribution of colloidal particles due to Brownian motion. Loss of aggregative stability leads to a coarse state, affecting sedimentation stability. Symptoms include turbidity, color change, and precipitation [14]. The attraction between nanoparticles will have a magnetic nature and change in line with the double electric layer dynamics. In this article, both aggregate and sedimental stabilities were investigated using dynamic light scattering, temperature stress tests, and turbidimetry.

Magnetic nanoparticle suspensions lose stability and monodispersity without stabilization, especially in the presence of coagulants. Steric stabilization occurs when surfactant molecules adsorb onto the surface of nanoparticles, preventing the formation of aggregates. This process creates a physical barrier preventing particles from sticking together, ensuring well-dispersed systems. Electrostatic stabilization occurs when charged particles repulse each other upon the formation of charges because of electrolyte ion adsorption or surface group dissociation [15]. The addition of multicharged ions with high adsorptive capacity contributes to the aggregation of nanoparticles due to changes in the surface charge and double electric layer. Therefore, only single-charged ions find use in electrostatic stabilization [16,17].

Steric stabilization is crucial for maintaining the stability of MNPs in biomedical applications. It relies on the density of the macromolecules on the surface of MNPs and the interaction of the stabilizing coating with the dispersion medium. Natural (dextran, alginate, chitosan, etc.) or synthetic (PEG, PVS, PVP, etc.) polymers are mainly used as stabilizing agents [18,19,20,21]. The effectiveness of steric stabilization depends on a homogeneous polymer coating and the presence of bridge effects, which can cause particle aggregation due to high-molecular-weight interactions [22]. Some polymer coatings are shown to decrease specific molecules’ adsorption [23]. Dextran is one of the more widely accepted polymers for particle modification because of its biocompatibility, variable size of its chains [24]. Even though dextran molecules are shown to desorb from particle surfaces, crosslinking agents may be used for polymer anchoring [25].

**Figure 1:**
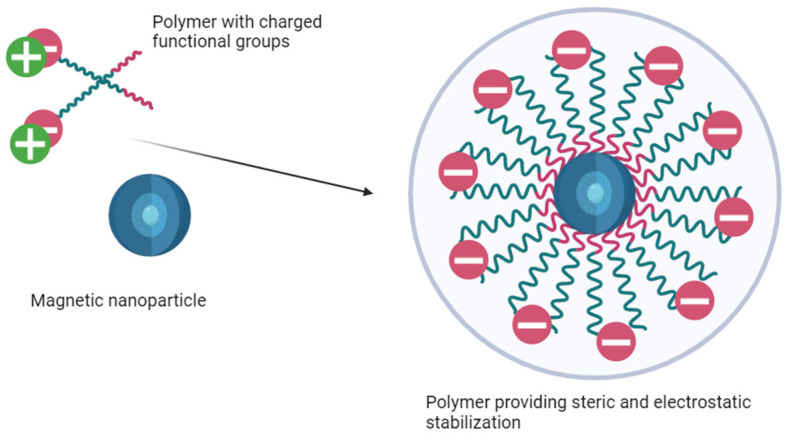
Electrosteric coating of nanoparticles.

A simple dextran coating can be switched to charged macromolecules or proteins to further stabilize suspensions [26]. However, this method is only effective at low ionic strength or pH, which differs from the isoelectric point of MNPs and proteins. The ionic strength of the medium determines the diffuse layer on the nanoparticles’ surface, preventing interaction and causing aggregation [27]. To obtain stable iron oxide nanoparticles both electrostatic and steric stabilizations (example in Figure 1) are necessary, as the isoelectric point of MNPs is pH 6.8 [28]. Compounds like polystyrene sulfonates, polyaspartate, polyacrylamide, polyacrylic acid, and carboxymethylcellulose are used as stabilizers [29,30,31]. A carboxymethyl-dextran coating may offer the best opportunities for application, as it combines the biocompatibility of dextran with high reactivity for modification. MNPs coated with carboxymethyl dextran exhibit superparamagnetism and stability, potentially serving as contrast agents in MRI [32].

Nanoparticles interact with cellular systems, which have varying media parameters that influence a magnetic suspension’s stability. Nanoparticles adsorb macromolecules upon contact, creating a protective coating called a corona [33,34]. This corona formation depends on the particle surface’s properties and can be controlled through modification, but then the size of the nanoparticle–corona complex also affects the biodistribution of the therapeutic composition [35]. Most up-to-date synthesis approaches, however, still achieve great applicability for nanoparticles [36].

A possible combination of steric and electrostatic stabilizations, as well as tackling unwanted protein sorption approaches, is coating or conjugating magnetite nanoparticles with BSA. Several commercially available nanoparticle preparations, as well as a number of agents undergoing preclinical research, have noted the use of albumin for stabilization [37,38]. For example, a magnetite–Au@BSA conjugate has been shown to retain a size distribution and morphology similar to pure magnetite nanoparticles [39]. Coating with BSA did not interfere with the superparamagnetism in a phantom MRI study. Similarly, BSA-TEOS-coated magnetite was used for four cycles of lysozyme extraction without changes in its physicochemical properties [40]. Many research groups have previously investigated SPION stability in the presence of serum proteins. In [41], the authors immobilized BSA on SPIONs intended for hyperthermia. Their findings suggest that BSA-SPION particles remain stable in water and PBS, and the passivation of the particle surface with protein prevents significant aggregation. Folic-acid-doped BSA also provides the possibility of the prolonged usage of SPIONs in targeted delivery [42], presenting high biocompatibility as well. Finally, the models presented in [43] emphasize the importance of balancing electrostatic and steric stabilizations to prevent particle clumping and surface ion chelation during in vivo application

While substantial research has been conducted, the question of the precise effect of albumin on the time intervals for using magnetite nanoparticles is still open to debate. In [44], the authors monitored the properties of stored SPIONs over a 12-week period, reporting substantial stability for dextran-coated preparations. As for an albumin corona, the protein coat lowers the unwanted uptake and acidic degradation of particles when assessed via an in vitro model [45]. In this study, the stability of MNPs in the presence of albumin was evaluated both under normal storage conditions and by modeling the use of particles in vivo. The goal was to formulate and offer a quick and available method of evaluating electrosterically coated SPIONs’ shelf life. To do this, we evaluated the colloidal stability of nanoparticles that interfaced with BSA (to mimic a stabilizer) or HAS (to mimic blood serum) in media with various pHs. Many methods for assessing colloidal stability have been described, such as UV/VIS absorbance, FTIR, and geometry and charge monitoring, all of which are time-consuming [46]. In this paper, for the first time, we adapt an accelerated aging method for characterizing colloidal stability, which makes it possible to evaluate the stability of nanosuspensions both under storage conditions and to simulate the stability of particles when absorbed by cells or introduced into the body.

## 2. Materials and Methods

### 2.1. SPIONs Synthesis and Characterization

SPIONs were prepared by coprecipitation from Fe^2+^ and Fe^3+^ (FeCl_3_·6H_2_O, FeSO_4_·7H_2_O, Vekton, Saint-Petersburg, Russia) salt solutions with 1:2 (0.3 and 0.6 mol/L respectively) molar ratio in nitrogen media at pH 10 and 80 °C, adapted from the Massart method [47,48]. The precipitation was performed by the dropwise addition of NH_4_OH (Vekton, Saint-Petersburg, Russia) to the iron salt solution under a nitrogen gaseous atmosphere with vigorous stirring. The magnetite formation follows the reaction shown below:

2Fe^3+^ + Fe^2+^ + 8OH^−^ = Fe_3_O_4_ + 4H_2_O.

After the addition of reductant, the magnetite crystal formation was completed by continuous mixing at 80 °C for 8 min in inert media. The resulting magnetite crystals were washed with water on a magnetic rack until reaching the neutral pH of media and then weighted.

Nanoparticles were stabilized by coating biopolymer carboxymethyl dextran with a dextran-to-iron weight ratio of 3:1 (CMDx, 15 kDa, Sigma Aldrich, St. Louis, MO, USA) upon mixing the NP suspension with the polymer solution for 5 min. The resulting nanoparticles were then pulse-sonicated at 22 kHz and 1 kW output power (6 times for a 5 min interval with a 3 min rest) and purified by magnetic separation to remove large multidomain ferromagnetic particles. Further centrifuge separation, membrane filtration, and dialysis were applied to produce the final monodispersion.

The concentration of Fe^+3^ ions in the NP suspension was measured by the thiocyanate method [49]. Briefly, diluted SPION samples of 0.1 mL were incubated for 10 min at 80 °C in highly acidic (HNO_3_, Vekton, Saint-Petersburg, Russia) media to convert all iron to Fe(III) form. Then, the volume of the sample was adjusted to 2.4 mL with water. The samples were then mixed with 0.8 mL of a 0.8 M solution of potassium thiocyanate (Lenreactiv, Saint-Petersburg, Russia), and the Fe(III) formed a red complex with the thiocyanate which could be measured by molecular absorbance at 575 nm.

Determination of the carboxymethyl dextran concentration was by the anthrone method. This method is based on the breakdown of complex carbohydrates into monosaccharides in an acidic medium, followed by their dehydration and the formation of hydroxymethylfurfural, which forms a bluish-green complex compound upon reaction with anthrone. Then, 550 μL of distilled water, 50 μL of the diluted particle solution, and 3 mL of 0.05% anthrone sulfate (Sigma Aldrich, St. Louis, MO, USA) solution were mixed. The samples were heated in a boiling water bath for 2 min and then cooled, with the solution acquiring an emerald color, and the control sample remaining yellow–green. In the colored solutions, the dextran concentration was determined spectrophotometrically at a wavelength of 625 nm against the control with water. The concentration of carboxymethyl dextran was 2–3 g/mL. Before use, the MNP samples were dialyzed.

A transmission electron microscope, a Libra 120 (Carl Zeiss, Oberkochen, Germany), was used to produce the micrographs of the CMDx-SPION clusters with and without HSA. The samples were placed on copper grids coated with formvar/carbon films (Electron Microscopy Sciences, Hatfield, PA, USA) and stained with a 1% aqueous solution of uranyl acetate. The analysis of the particle size was performed using ImageJ software (National Institutes of Health, Bethesda, MD, USA; version 1.54d) to estimate the cluster areas and GraphPad Prism software (GraphPad Software, La Jolla, CA, USA; version 9.5.1.) to plot the distribution of cluster areas across samples. Each distribution included 70 clusters of varying sizes, selected randomly from multiple TEM images to ensure representativeness. The cluster areas were calculated in nm^2^ and scaled by ×10^−3^ for graphical representation to enhance clarity.

The hydrodynamic size and zeta potential of the SPIONs were assessed via dynamic light scattering (DLS) using a Zetasizer Pro (Malvern Instruments, Malvern, UK). The zeta particle potential was determined by applying an electric field in a standard spectrophotometer cuvette. A suspension (3 mL volume) was placed in a glass cuvette and treated by coherent laser radiation (633 nm wavelength), and the power of the light field scattered by the sample was recorded at an angle of 176 degrees using a photoelectric multiplier, ADC, and computer. The autocorrelation function of the light intensity was analyzed according to the standard device protocol (cumulant procedures). An experimental autocorrelation function was processed as a sum of the exponentials and calculated by the translational diffusion relation based on Stokes law. To prevent multiple light scattering, the suspension was diluted up to 50 times to reach the needed rate of homogeneity in the cuvette. Each measurement was performed with three repetitions. The Zetasizer Pro uses a combination of laser Doppler velocimetry and phase analysis light scattering (PALS) in a patented technique called M3-PALS to measure the particle electrophoretic mobility. The Zetasizer Pro software (ZS Xplorer; version 3.00) produced a frequency spectrum from which the electrophoretic mobility and, hence, zeta potential was calculated (with built-in software), implementing the Smoluchovsky model and Henry equation. The data were interpreted in terms of hydrodynamic diameter, polydispersion index, and Zeta potential. The size of protein corona was estimated by DLS using a comparison of the control and MNPs after heating at 70 °C in the range of 20–30 nm.

### 2.2. Albumin Fluorescence

A Varioskan Lux (Thermo Scientific, Waltham, MA, USA) reader was used to measure the fluorescence intensity of the complex of studied proteins with magnetic nanoparticles. By registering the temperature dependence of the relative intensity of the fluorescence of the proteins with the addition of SPIONs and calculating the fluorescence quenching constant, it is possible to deal with the nature of the interaction of the SPIONs and proteins (albumin from bovine and human sera, Sigma). A set of samples was prepared from a suspension of SPIONs-CMDx nanoparticles with different concentrations (by the Fe^3+^ content), as follows: 0, 0.028, 0.056, 0.084, 0.112, 0.14, 0.168, 0.196, 0.224, and 0.252 μM in 0.1 M PBS (Biolot, Saint-Petersburg, Russia) at a pH = 7.4, 5.5, or 4.5. The acidity of the buffer was set to represent normal blood plasma of an endosome and lysosome interior [50]. The sample also contained 5 mM BSA or HSA to mimic blood serum [51].

Measurements of the albumin fluorescence were performed at an excitation wavelength of 280 nm, an emission wavelength of 340 nm. The measurements were carried out at 25 and 36 °C.

An evaluation of the fluorescence quenching constant was performed according to the formula for the relative fluorescence intensity (RFI), as follows:

$$\frac{F_{0}}{F}=1+k\times [Q]$$

where F_0_ is the value of the protein fluorescence intensity (BSA or HSA);

F is the value of the fluorescence intensity of the protein complex (BSA or HSA) when nanoparticles are added;

k is the quenching constant of protein fluorescence (BSA or HSA), mol^−1^;

Q is the concentration of magnetic nanoparticles, μM.

### 2.3. SPIONs’ Stress Test

For the rapid assessment of the colloidal stability of SPIONs in storage conditions and in the presence of serum albumin, the technique of thermally induced accelerated “aging” was used, since without heating, the degradation and aggregation of NPs occur rather slowly. The methodology is based on the work of Rabel et al. [52]. However, several modifications were introduced. An assessment of the colloidal stability and a stress test were performed, which reduced the testing time from 24 h, as proposed by M. Rabel, to 2 h. A temperature of 70 °C was chosen to reduce the testing time (maximize the speed of possible reactions) when heating the composition of the nanoparticles with albumin, without causing structural changes. The possibility of assessing the colloidal stability and stress testing in the presence of HSA is demonstrated. The applicability of the temperature-adjusted storage time calculation method is also demonstrated for the case of a protein crown made from HSA.

In general, 50 mL of 0.1 M PBS (pH 7.4, 5.5 or 4.5) containing 50 μg of SPIONs, by the Fe^3+^ content, was filtered (0.22 μm PVDF) onto a sterile glass. After filtration, the resulting suspension was stirred in a 1000 min^−1^ shaker for 30 s. Then, 1.2 mL of liquid was taken from the resulting solution into 12 Eppendorf-type test tubes for the stress-test experiment, as follows:

Test tubes with the SPION/protein suspension were placed in a water bath (ICC basic pro 20, IKA, Staufen, Germany), where samples were heat treated at 70 °C for 120 min. The samples were taken after 30, 60, and 120 min, with an additional control sample that did not undergo heating. Then, 500 µL of each suspension was taken to determine the aggregate colloidal stability of the SPION clusters by DLS. The remaining suspension was centrifuged at 12,000 min^−1^ for 5 min. Then, 100 µL of the supernatant was taken for the determination of the iron content.

Similar experiments were carried out at a concentration of 0.05, 0.5, and 1.0 mg/mL HSA in a 0.01 M PBS.

### 2.4. SPIONs’ Age Test and Shelf Life

To verify the results obtained in the stress test, the SPION suspensions containing 0.5 mg/mL HSA were subjected to long-term storage.

Likewise for the stress test, an aqueous suspensions of SPIONs in 0.1M PBS containing 0.05, 0.5, or 1.0 mg/mL of protein was added to sterile Eppendorf-type tubes, which were stored at a temperature of 8 °C.

Samples were taken for evaluation after 1, 30, 90, and 120 days of storage, as follows: 500 µL of suspension for DLS; the remaining suspension was centrifuged at 12,000 min^−1^ 5 min, and 100 µL of the supernatant was taken for colorimetry.

To clarify the temperature coefficient, the results of the accelerated “aging” and long-term storage of nanoparticles with protein (HSA content of 0.5 mg/mL) were compared. To differentiate among the stages of nanoparticle aging, we evaluated regions of unidirectional (decrease-only and increase-only) changes in the parameters of the suspension. Combining the tendencies of the hydrodynamic diameter and Fe ion concentrations in both the stress and age tests, we determined an approximate shelf life for a magnetic nanosuspension at 70 °C. The shelf life for other temperatures was considered according to the following relationship [53,54]:

$$C=A^{\frac{t_{exp}-t}{10}}\times C_{exp}$$

where C is the shelf life of SPIONs at temperature t; C_exp_ is the shelf life of SPIONs at t_exp_ in the stress test; and A is the temperature coefficient.

The validity of using Van’t Hoff’s rule was confirmed by calculating the activation energy at the following two temperatures: 8 °C and 70 °C. The fact that the activation energy and temperature approximately satisfy the relationship, taking into account the Arrhenius rate constants, confirms that the Van’t Hoff rule can be used in this case (when assessing the effect of temperature on the rate of change in the iron content in the supernatant), as follows:

$$E_{A}=\frac{{RT}^{0}}{10}$$

$$E_{A}=\frac{RT_{1}\times T_{2}}{T_{2}-T_{1}}\times ln\frac{k_{2}}{k_{1}}$$

The data used in the calculation included R = 8.31; T_1_ = 70; T_2_ = 8; k_2_ = 0.1583; and k_1_ = 0.3159 for the unimodal stages of the suspension’s aging.

### 2.5. SPIONs NMR

The 1H NMR spectra and proton relaxation times were measured on a Spinsolve 60 spectrometer (Magritek, Aachen, Germany). The samples, in an aqueous suspension, were placed in a cylinder ampoule with a 5 mm diameter. The spectra were recorded at an ambient temperature without rotating the sample. The TMS sample was used as an external standard (0.0 ppm) for measuring the chemical shift. When recording the spectra, the one-pulse program was used, the number of accumulations was 5, and the delay time between pulses varied in the range of 2–5 s. Relaxation times T_1_ and T_2_ were measured using inversion recovery and the Carr–Purcell–Meibom–Gilles sequence according to a common device protocol.

### 2.6. Turbidimetry

**Figure 2:**
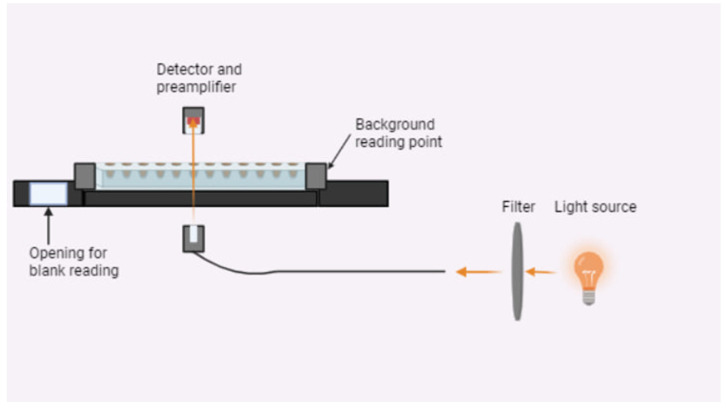
Turbidimetry experiment scheme.

The kinetic stability of a magnetic nanoparticle suspension in the presence of BSA, the HSA was studied using a turbidimetric method. Turbidimetric data on the kinetic stability of the SPION/protein suspension (light transmission in a colloid media) were measured over 0–5 days at 8 °C. Light absorption, including the contribution of multiple static light scatterings from aggregates, was measured on a Multiscan photometer (Thermo Fisher, Waltham, MA, USA) equipped with a laser operating at 405, 450, and 620 nm. For the optical density measurements, 96 plate cells were used. Light intensities were recorded at a 180° angle and 5 sec of pulse shaking after the preliminary vortexing of a 1 μL sample for 1 min. A general scheme of the experiment is shown in Figure 2.

A significant contribution to the weakening of the intensity of the light that passed through the analyzed dispersed system is made by the following two types of interaction of electromagnetic radiation with matter: light absorption and light scattering on particles. The law of change in the intensity of the incident light can be expressed as follows:

$$I=I_{0}\times e^{\left(-\tau l\right)}$$

where I_0_ is the intensity of the incident light;

τ is the turbidity;

l is the length of the path that passes by the light flux.

The ability of the studied system to scatter light is characterized by the turbidity coefficient, which is a product of the number of particles, n, in it and the scattering cross-section of particles, C:

τ=n×C

The scattering cross-section of particles is an integral of the scattered light in all directions relative to the center of the particle. This parameter is related to the geometric cross-section of the particle through the scattering coefficient, Q, as follows:

$$C=Q\pi d^{2}$$

where d is the particle diameter.

To avoid multiple light scattering, conditions of low concentrations were created, amounting to 50 μg Fe(III)/mL, at which each particle could be considered as an independent source of light scattering.

### 2.7. Statistics

The statistical evaluation was performed in Graphpad Prism software (version 9.5.1). For the statistical analysis of the parametric Student’s *t*-test was employed for independent samples and parallel measurements. The significance level was equal to α = 0.05 for all tests. The confidence intervals were at the 95% level.

## 3. Results

### 3.1. Synthesized Nanoparticles’ Characteristics

**Figure 3:**
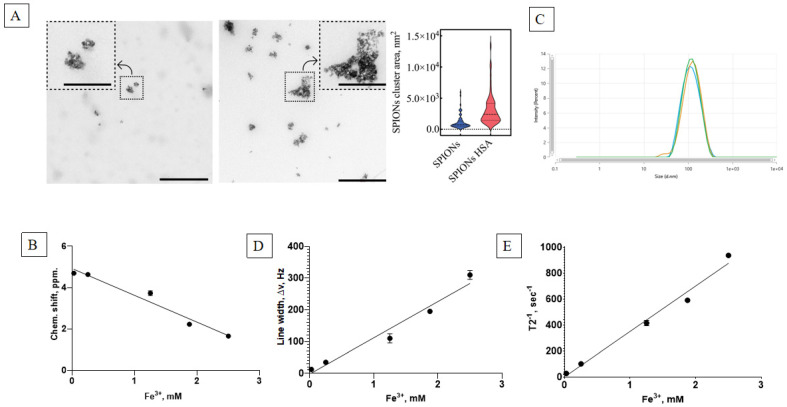
Physicochemical characterization of the magnetic nanoparticles. (**A**) TEM images of CMDx-SPION clusters without (left panel) and with HSA (center panel). The right panel presents the distribution of cluster areas (scaled by ×10⁻^3^ nm^2^ for graphical clarity). Scale bars: 500 nm for the main images; 200 nm for insets. The violin plot shows the median cluster areas (dashed lines) and interquartile ranges (25% to 75%, dotted lines), illustrating the central tendency and variability in cluster sizes. The width of each distribution in the violin plot reflects the density of data points in different cluster areas, with broader sections indicating a higher concentration of values. (**B**) Chemical shift dependence on the SPION concentration in water. (**C**) Size distribution of the nanoparticles. (**D**) Line width dependence on the SPION concentration in water. (**E**) Relaxation time of the SPION water suspensions.

The initial studies of the physicochemical properties of the synthesized SPIONs show that the suspension consisted of highly dispersed ferrofluidic material with an ability to concentrate in a constant magnetic field and to later be resuspended immediately upon stirring or dilution. The TEM analysis revealed the formation of SPION clusters with varying areas, as previously described. To assess the distribution area of these clusters, both in the presence and absence of HSA, 70 randomly selected clusters were analyzed for each condition, with samples taken from multiple TEM images to ensure representativeness. The distribution plot (Figure 3A, right panel) demonstrates that the presence of HSA induced a shift in the cluster area, with the median value increasing from 738.5 nm^2^ (without HSA) to 2422.1 nm^2^ (with HSA). This corresponds to an approximately 3.3-fold increase in the median cluster area, indicating that albumin promotes SPION aggregation. The interquartile range (25% to 75%) also broadened, extending from 507.9 to 1313.5 nm^2^ in the absence of HSA to 1453.1 to 4142.8 nm^2^ in its presence, reflecting an increased variability in cluster sizes upon HSA’s addition. These findings suggest that HSA plays a significant role in modulating SPION aggregation behavior (Figure 3A).

Synthesized suspensions had a high Fe ion content, as measured by the colorimetric method. The dynamics of the chemical shift (Figure 3B) and resonance line widening (Figure 3D) corresponded well with the superparamagnetic material. The NMR relaxometry studies show a linear dependence of the main magnetic parameters of the nanoparticle concentration, with a low T_2_ relaxation time of ~1 ms and a high relaxation effectiveness (Figure 3E). The stable nanosuspension had a major dextran-coated nanoparticle fraction of 100 nm in size (Figure 3C).

### 3.2. Albumin Fluorescence Assessment

A Varioskan Lux reader was used to measure the fluorescence intensity of the complex formed between the studied proteins and magnetic nanoparticles. The temperature dependence of the relative fluorescence intensity of the albumin in the presence of SPIONs is used to determine the quenching constant for fluorescence.

**Figure 4:**
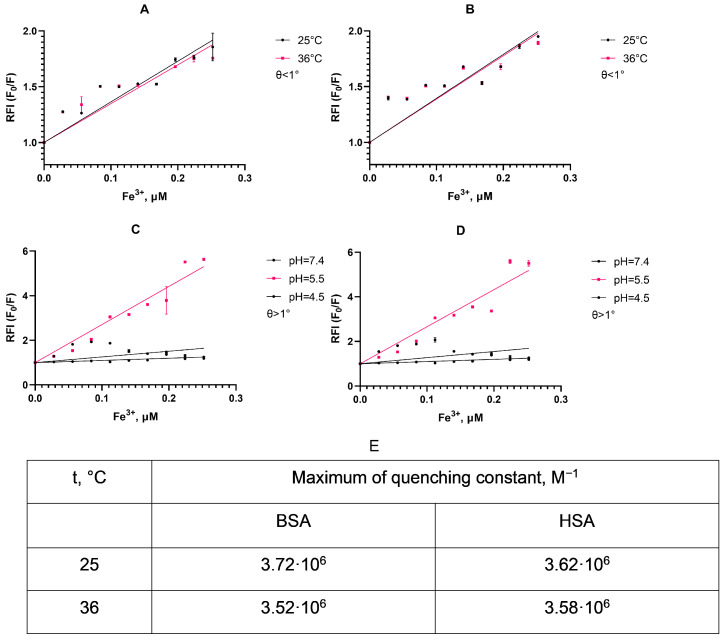
Analysis of the albumin–nanoparticle interactions according to the dependence of the albumin fluorescence intensity (340 nm) on the Fe^+3^ content in SPIONs: (**A**,**B**) fluorescence intensity of the SPIONs with BSA and HSA, respectively, in PBS; (**C**,**D**) fluorescence intensity of the SPIONs with BSA and HSA, respectively, at a set pH; (**E**) calculated quenching constants.

As can be seen from the displayed plots (Figure 4A,B), the slope of the linear approximation for the fluorescence quenching differs non-significantly depending on the temperature in accordance with a static mechanism for quenching albumin fluorescence [55]. This suggests a simple protein corona formation between albumin and SPIONs, as a stable low-fluorescence complex between particle surface and protein is formed upon interaction without the dependence of fluorescent amino acids in a deeper protein structure. We also detect C_50_ for the protein quenching at a SPION concentration of 0.252 μM, corresponding to approximately half of the protein particle population in a suspension with a corona coat.

As we traced the stability of this protein corona (Figure 4C,D), the quenching of the fluorescence intensity of the HSA is pH-dependent. Thus, at pH 5.5, the relative fluorescence intensity increases quite rapidly with an increase in the concentration of nanoparticles compared to other pHs. At pH 4.5, fluorescence quenching practically did not occur, which may be due to the proximity of this pH to the isoelectric point of the HSA (pI 4.7). Additionally, the similarity of the quenching constants may suggest that, in both albumin types, a fluorescent complex with a similar emission lifetime formed with no rapid dynamics in molecule conformation [56].This characteristic is shown both at 25 and 36 °C, and indicates an instability of the protein corona upon SPION uptake in cells, as endosomes and lysosomes have a lower pH.

### 3.3. Changes in SPION Parameters upon Albumin Incubation

**Figure 5:**
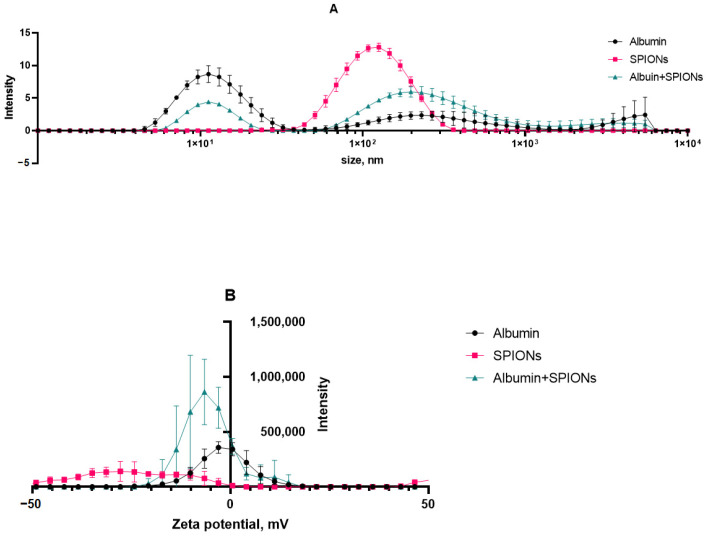
DLS results for SPIONs upon incubation with albumin: (**A**) size distribution; (**B**) zeta-potential distribution.

The size and charge distributions of aqueous suspensions of SPIONs in the presence of albumin are illustrated in Figure 5. Based on the DLS analysis, the first peak in the figure can most likely be attributed to albumin nanoparticles with the size of ~10 nm. The observed polydisperse fractions belong to aggregates of protein in the sample media. The prepared SPIONs, however, showed a monodisperse character, with a 107 nm average size (by peak intensity) and a PDi of 0.170. The majority of the particle population had a surface charge of −28 mV. Upon incubation with albumin and protein corona formation, we observed that the size of nanoparticles inherited a multimodal distribution of albumin, with the main peak being close to 200 nm. This result was further affiliated by a shift in the polydispersity index of 0.17 (SPIONs) to 0.93 (SPIONs/albumin). At the same time, a protein corona seemed to neutralize most of the negative surface charge of SPIONs.

### 3.4. Turbidimetric Analysis of the SPION Suspension

**Figure 6:**
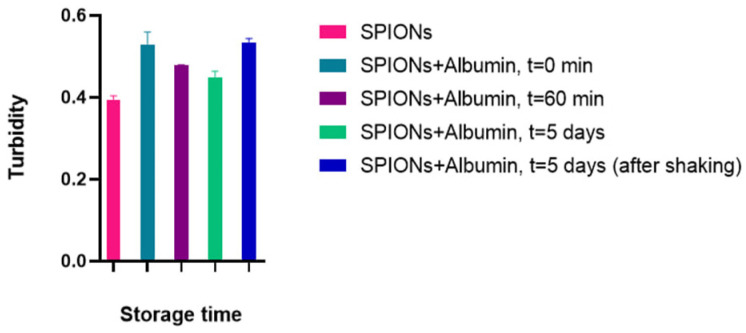
Turbidity of the magnetic suspension of SPION/albumin at a light absorbance of 405 nm after a 2 h treatment at an ambient temperature of 22–25 °C after 5 days of storage.

With constant parameters for the number of particles and the path length of the light beam, the change in the turbidity coefficient’s value is caused by a change in the scattering cross-section of the particles. Moreover, the turbidity value should increase proportionally to the increase in the effective hydrodynamic radius of the particles upon their aggregation. Pure albumin, here, exhibited no change in behavior depending on the concentration (overlapping data at 0.5 and 0.05 μg). Proceeding from a very low index change during the experiment (from 0.52 to 0.51, ~2% for SPIONs with 0.5 mg/mL albumin), we can assume the kinetic stability of the corona-coated nanoparticles with no significant cluster growth (Figure 6). Thus, the recording turbidity coefficient allows for an evaluation of the dispersion of the system, and in the kinetic monitoring mode, it is possible to track changes in the studied system over time.

### 3.5. Particle Aging Dynamics

**Figure 7:**
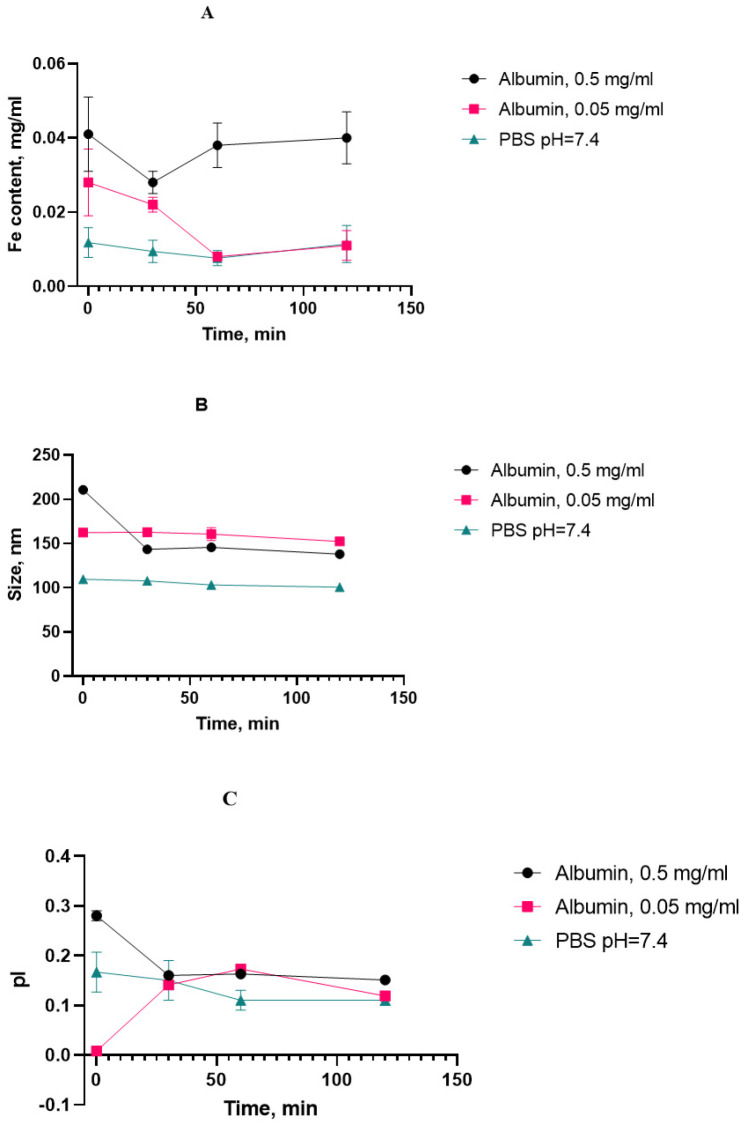
Physicochemical parameters of the SPIONs in the stress test: (**A**) Fe^3+^ content; (**B**) hydrodynamic diameter; (**C**) polydispersity index. n = 3 (for each parameter).

Monitoring the properties of SPIONs during accelerated aging in the presence of albumin at 70 °C, we obtained a set of dynamics of interest, as presented in Figure 7.

**Figure 8:**
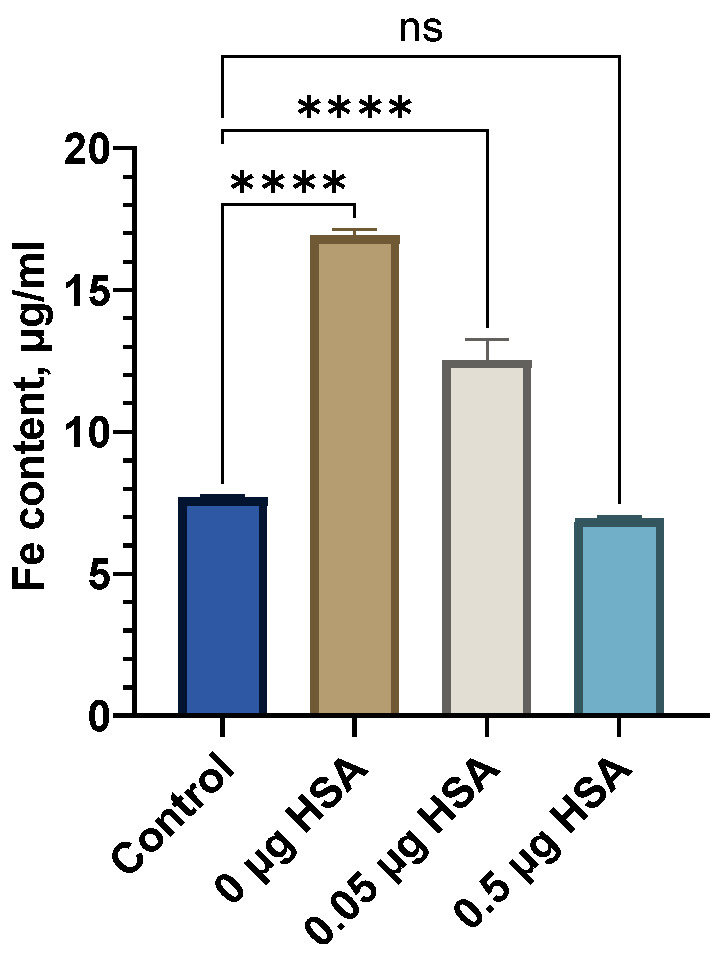
Iron content in the SPION sedimentation test after heating with HSA. ****—p < 0.0001; ns—non-significant.

We initially chose the iron content in the supernatant after the suspension’s centrifugation as a “stress marker” to quantify the amount of iron degrading from nanoclusters upon aging. With the increased presence of HSA, the concentration of iron in the supernatant did not increase with the heating time compared to the control point (without heating), and it can be concluded that the sedimentation stability of the system was maintained under the experimental conditions (Figure 8).

The protein corona between albumin and SPIONs can be calculated as the difference between the sizes with and without albumin after heating for 120 min. A layer of approx. 20–30 nm was formed by the static mechanism.

**Figure 9:**
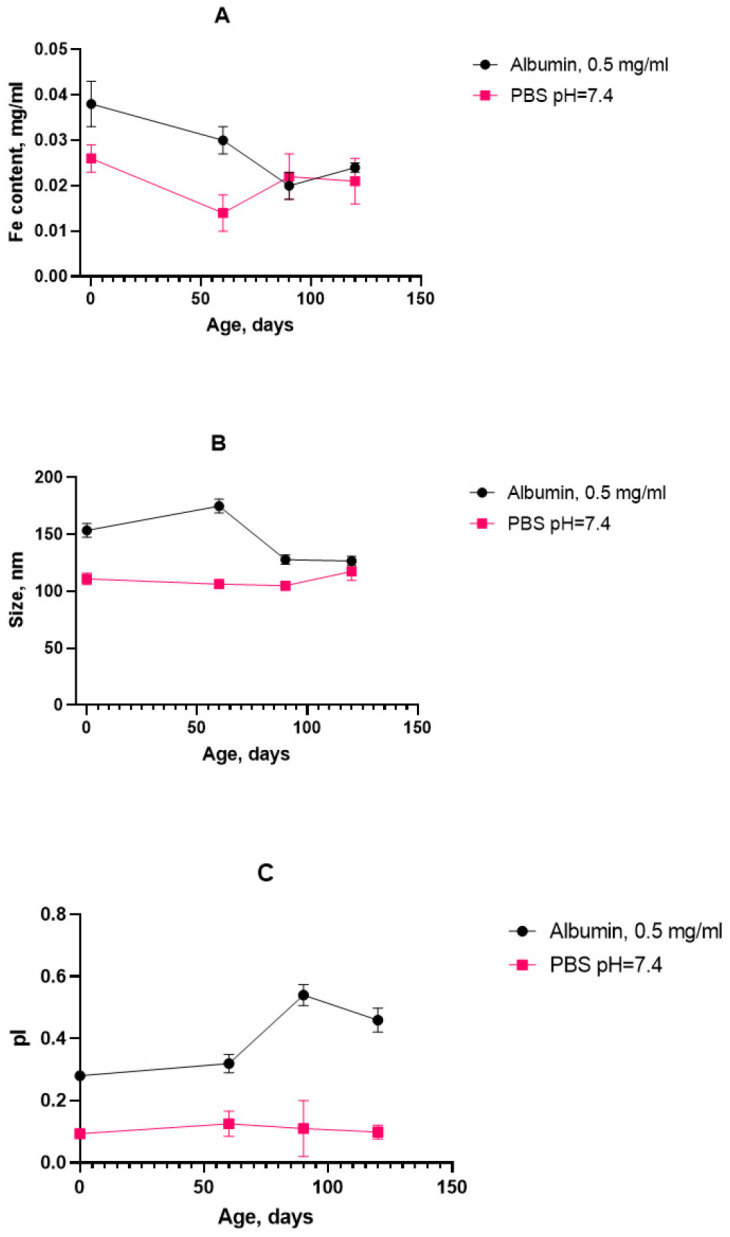
Properties of SPIONs during prolonged storage: (**A**) Fe^3+^ content; (**B**) hydrodynamic diameter; (**C**) polydispersity index. n = 3 (for each parameter).

As for the aggregation stability, the obtained results indicate that the SPION suspension underwent no significant aggregate formation during the course of the stress test. The decreasing tendency in the growth of the cluster diameter can be attributed to a decrease in the double electric layer and degradation of the dextran coat. Since there is also a decrease in the polydispersity index, it can be concluded that the particle size distribution becomes more uniform and the system becomes monodisperse, hence less prone to aggregation. On the basis of the high rate change in the physical properties of the magnetic nanosol at an HSA concentration of 0.5 mg/mL, this SPION/HSA suspension was chosen for long-term storage. The size, polydispersity index, and iron content in the supernatant were obtained after 4 months of storage of the magnetic nanoparticles (Figure 9).

The graph shows that the iron concentration did not exceed the initial value over the storage period, and it also reached a plateau, which confirms the sedimentation stability of the system. The particle size seemed to increase slightly during the measurement period, but then decreased and stabilized. This indicates the absence of the formation of large aggregates. The dynamics of the time evolution correspond to the stress tests. However, in the case of the prolonged storage of the SPION suspension with albumin, there were features of significant shifts in the polydispersity index, which may indicate uneven protein adsorption on the surface of nanoparticles.

**Figure 10:**
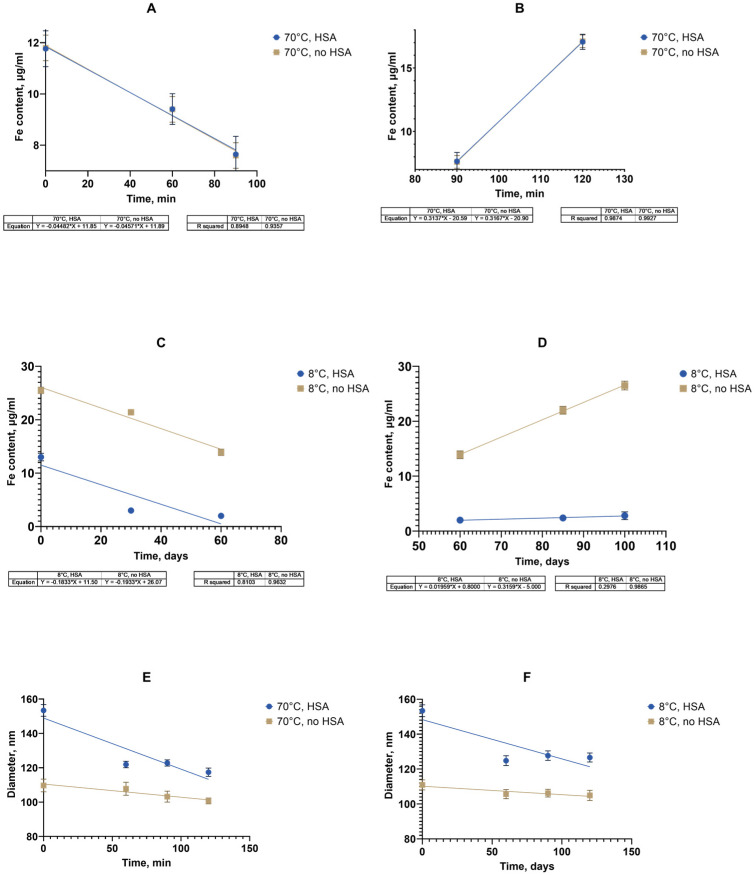
Storage stability of SPIONs in the dispersion during long cold storage (8 °C) and after short temperature stress action (70 °C) in the presence albumin: (**A**,**B**) Fe^3+^ release in degradation after temperature stress; (**C**,**D**) Fe^3+^ release in degradation during long storage at 8 °C; (**E**,**F**) nanocluster size dynamics for short stress and long storage, respectively. Control sample—no albumin added.

During the storage period’s analysis and calculation, based on the results obtained in the process of the “accelerated aging of MNPs” (2 h at 70 °C) or accelerated “aging” with a stress test, it was possible to estimate the time during which controlled quality indicators (size, PDI, and Fe(III)) remained within acceptable limits. An approximate relationship of the Van’t Hoff’s rule of about a 2–4-fold increase in the rates of chemical reactions with an increase in temperature by 10 °C was used to predict this time. For the initial determination, a coefficient of 2.5 was used as the common case for the shelf-life calculations [57,58]. The experimental value of the temperature coefficient was determined based on the data from the accelerated “aging” at 70 °C and a pH of 7.4 with the stress test and long-term storage with a stress test (Figure 8 and Figure 10). The data on the linear approximation of the unidirectional changes in the iron concentration in the supernatants were compared (Figure 10A–D).

The figure shows the linear approximation data for the change in the iron concentration of the supernatant. The time for a 25% decrease in the iron concentration during the first stage was calculated using the linear approximation data on the dependence of the iron content on the supernatant over time. The time taken for the concentration to decrease to 9 μg/mL was 38.9 min. In accordance with Van’t Hoff’s rule using a coefficient of 2.5, the forecast for the time to achieve a similar concentration at 8 °C was calculated, which was 7.9 days. The experimental determination of the time to achieve a 25% decrease in the iron concentration in the supernatant in accordance with the linear approximation data during storage at 8 °C was 32.9 days. In accordance with the obtained experimental value, a coefficient of 2.5 was adjusted to 3.15 by the selection method (2.6–10.1, 2.8–16, 3.0–24.5, and 3.15–33.1). The temperature coefficient for the second stage was similarly checked with a 25% increase in the concentration and its value was determined to be 3.15. Thus, it was shown that it is possible to use the adjusted temperature coefficient of 3.15 to calculate the shelf life of a suspension of nanoparticles coated with carboxymethyl dextran based on the data for the entire period of the accelerated “aging” of two hours followed by a stress test. The shelf life of a suspension with known characteristics at 8 °C was 102.4 days (3.4 months), at 25 °C, 4.7 days; at 37 °C, 1.2 days. In the case of the accelerated “storage” of particles in the presence of albumin with subsequent stress testing, since a decrease in the rate of iron release into the supernatant was observed depending on the concentration of the HSA (Figure 9), up to its stabilization at an HSA of 0.5 mg/mL. The conditions for calculating the temperature coefficient and its refinement were insufficient. It is possible that a longer observation period than that used in the experiment (3–4 months) is required to register changes in this case. However, a change in the size of nanoclusters in the presence of albumin both during accelerated “storage” and long-term storage at 8 °C was recorded (Figure 10E,F).

The temperature coefficient of the process was estimated and refined taking into account the data obtained for nanoparticles without albumin in the medium. The time for a 10% decrease in the iron concentration was calculated using a linear approximation of the dependence of the hydrodynamic diameter of nanoclusters on time during accelerated “storage”. The time taken for the size to decrease to 138 nm was 37 min. In accordance with Van’t Hoff and using a coefficient of 3.15, the forecast for the time to achieve a similar concentration at 8 °C was calculated as 31.6 days. The experimental determination of the time to achieve a 10% decrease in the iron concentration in the supernatant according to the linear approximation during storage at 8 °C was 120.5 days. In accordance with the obtained experimental value, the coefficient of 3.15 was adjusted by the selection method to A = 3.91 (3.8–101, 3.9–118.7, and 3.91–120.6). The shelf life of the suspension with known characteristics at 8 ⁰C was 120.6 days (4 months); at 25 °C, 11.9 days; and at 37 °C, 2.3 days. To understand the influence of the HSA, the temperature dependence of the hydrodynamic diameter of the nanoclusters on the time at the two temperatures of 70 °C and 8 °C was determined in the absence of albumin using the algorithm described above, with A = 3.32.

The shelf life of a suspension with known characteristics without albumin at 8 °C was 80 days (2.7 months); at 25 °C, 10.3 days; at 37 °C, 2.5 days.

Thus, the approximate shelf life for the SPIONs without and with HSA present were 80 and 121 days, respectively, at 8 °C. Based on the data from accelerated “aging” for two hours at 70 °C, it became possible to predict the characteristics of the nanoclusters for a period of 3–4 months for various experimental or storage temperatures.

## 4. Discussion

This study investigated magnetic nanosuspensions, which are unstable systems that may be prone to degradation during storage. The study investigates a nanodispersion of magnetic iron oxide particles of SPION/albumin, focusing on the sedimentation and aggregation properties.

**Figure 11:**
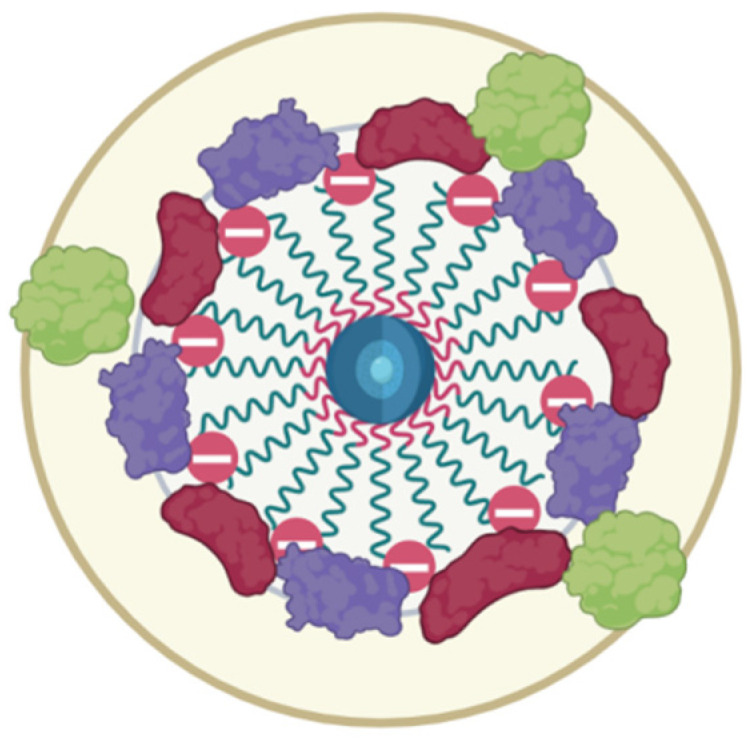
Schematic view of the sera protein (including albumin) corona around magnetic iron oxide nanoparticles stabilized by carboxymethyl dextran.

The initial characterization of the synthesized nanoparticles confirmed the yield of superparamagnetic clusters of less than 200 nm in size (Figure 3). These particles adhere to the usual desired parameters for theranostic nanocarriers. Further study revealed a stable protein corona formed from albumin’s interaction with the magnetic nanoparticles. The fluorescence analysis revealed a static fluorescence quenching mechanism, forming a stable SPION/albumin complex (Figure 4). The study also found that nonradiative transfer does not affect the deep amino acid residues of Trp and Tyr inside the protein corona. These findings are similar to those for citrate BSA/SPIONs with a dense monolayer of protein [59]. The heated albumin itself contains monomers and oligomers or aggregates. In addition to the native protein, the heterogenous fraction presents higher sedimentation coefficients [60]. This may lead to different behavior by MNPs covered with CMDx during heating (colloid solution and colloid sol). The carboxymethyl dextran shell easily adsorbs albumin and forms a hybrid coat layer. As opposed to citrate SPIONs, the ensured coat stabilization was provided by H-H bonds between saccharide residues and magnetite Fe domains. The zeta potential of such carboxymethyl dextran SPIONs under the influence HSA take on a neutral value (Figure 5). The latter leads to weak adherence between magnetic nuclei and nonpacked metastable clusters in an HSA saturation state on the surface. The presented model of this protein–SPION interface can be viewed in Figure 11. This general scheme matches well with the results of the structural analysis of SPIONs modified by blood sera proteins with magnetite nuclei and albumin [61].

The obtained DLS results and estimated Fe ion content in the stable phase indicate that the adsorption of albumin on SPIONs may be accompanied by destabilization of magnetic flocculated clusters. According to the results of the DLS study, SPION/albumin nanoparticles coexist in the suspension as individual, separate, and stable particles and in the form of weakly adhered metastable clusters. These clusters are stabilized by van der Waals attraction and magnetic forces when placed in an external field. The multimodal fraction dispersion size function, as measured by light scattering, is evidence of such a structural arrangement. The size of the magnetic nanoparticles representing the dispersed phase of the colloid under study is comparable to the wavelength of the acting radiation, which can lead to light interference and a decrease in the scattering intensity, but the phase difference leading to this effect is completely compensated when registering a signal at an angle of zeros. This case is realized by applying the turbidimetric method of analysis. The turbidimetric approach allows us to use more simple phenomenological relations derived for particles whose size is smaller than the wavelength of light (Figure 6).

The bare SPIONs stabilized by carboxymethyl dextran were characterized in the monodispersed mode with a 100 nm range; The satisfactory stability of the suspension was achieved by the generation of a surface charge of −28 mV. The prepared albumin solution has a monomodal view, where the first peak in the light scattering plot is attached to albumin associates with a size of ~10 nm. In the concentrated protein solution, we observed a polymodal size dispersion with the appearance of nanoaggregated protein species, with an average size of 200–300 nm. Upon addition of albumin to the SPION suspension and following the formation of the protein corona, the size distribution function of the nanoparticles became multimodal, which resembled albumin’s characteristics, with the main peak of the light scattering being typical for spheres with a size close to 200 nm. According to our findings, the protein albumin corona neutralizes most of the negative surface charge of the SPIONs but does not induce the falling out of magnetic material to a notable degree for a long period of time (Figure 8). The latter provide long-term colloid stability of SPION/HSA in storage (8 °C) for 6 months without phase separation (Figure 7 and Figure 9).

The rapid temperature stress aging approach was tested for the case of magnetic nanosol stabilized by HSA protein. To reduce the amount of time taken to assess the colloidal stability of the magnetic nanoparticles in combination with human serum albumin (HSA) at a concentration of 0.5 mg/mL in a phosphate buffer from 6 months to several days, a heat-induced accelerated “aging” method was for the first time introduced for electrosterically stabilized magnetic nanoparticles (Figure 7 and Figure 9). The accelerated stress-aging method was previously suggested as the cutting time for the evaluation of low-molecular pharmaceutical substances in solution [62,63]. The temperature stress-aging approach has not previously been applied in the case of the magnetic iron oxide nanoparticles with carboxymethyl dextran. The peculiar feature of the studied magnetic suspension is the usage of HSA compared to the usual BSA-only study [64]. The calculation of the storage time based on the temperature coefficient for compositions with nanoparticles as for pharmaceutical substances is proposed. An amendment was introduced to calculate the temperature coefficient based on the comparison of the actual and estimated storage times at a temperature of 8 °C. The initial stabilization of the magnetic nanoparticles was achieved by coating with carboxymethyl dextran, with the subsequent addition of titratable amounts of albumin. Albumin has a heat conformational transition that can be taken into account. The reliability of the results of the accelerated stress-aging was controlled comparing long-term storage of the magnetic suspension at 8 °C. To assess the aggregation’s stability, the size and polydispersity index of the particles with protein were measured using dynamic light scattering and electrophoresis. The sedimentation colloidal stability of the suspensions was estimated by the iron (III) content in the supernatant obtained after sedimentation of the particles in the centrifugal field of the centrifuge. It was found that the general monitoring of the colloidal stability parameters of a magnetic suspension in the presence of albumin reveals a correlation between aggregation and sedimentation stability. After the long-term storage of the magnetic suspension sediment, some deposits consisted of metastable nanoclusters. The metastable nature of the deposit was confirmed by a mild shaking of the vessel containing dispersion. Shaking transforms the deposit into a stable magnetic nanosuspension with the initial DLS parameters of the DLS measurement monitored before storage (Figure 10). The distribution pattern of the nanoparticles in a complex with protein during the accelerated aging was influenced by the presence of albumin, a key factor in the production of a metastable magnetic composition. This stability of the suspension and SPION/albumin allows for the consideration of the magnetic complex as an independent carrier. This suggests that albumin could be used as an additive for nanosuspensions of carboxymethyl-dextran-coated SPIONs, as well as ensures that administered nanocarriers might not immediately aggregate in blood flow. A decrease in the stability of the magnetic complex of albumin when lowered to pH = 4 should be taken into account when nanoparticles are used to deliver therapeutic agents to target cells, as following the disintegration of the magnetic nanocarrier in the endolysosomes, the release of the drug into the cytoplasm occurs. The magnetic carrier based on carboxymethyl-dextran-coated magnetite nanoparticles retained the necessary stability for the targeted delivery of therapeutic agents during parenteral administration in the presence of serum albumin.

## 5. Conclusions

The method of accelerated temperature stress “aging” of nanosized iron oxide suspensions is efficient for the rapid assessment and prediction of colloidal stability over time of nanosized magnetic iron oxide suspensions stabilized by albumin. The express approach is achieved by applying a high stress temperature of 70 °C and using the albumin’s influence on the stability of the iron oxide nanosol. According to the fluorescence quenching and DLS data, colloidal stability is ensured by the formation of an albumin corona and clustering of SPIONs/albumin. The formation of magnetic nanoparticle clusters with a protein corona affects the sedimentation stability of the colloidal system under physical influences (temperature and sedimentation by a centrifugal field). The temperature stress approach is appropriate for the prediction of the colloidal stability of nanosized magnetic iron oxide suspensions over long periods, which was shown by the assessment of the shelf life of iron oxide dispersions with a therapeutic iron content of 50 μg/mL and albumin in the range from 0.05 to 0.5 mg/mL.

## Data Availability

The datasets used and/or analyzed during the current study are available from the corresponding author Maxim Shevtsov upon reasonable request.

## Abbreviations

MNP	Magnetic nanoparticle
SPIONs	Superparamagnetic iron oxide nanoparticles
BSA	Bovine serum albumin
HSA	Human serum albumin
CMDx	Carboxymethyl dextran
DLS	Dynamic light scattering
TEM	Transmission electron microscopy
PBS	Phosphate-buffered saline
RFI	Relative fluorescence intensity
